# A Novel Investigation of a Blister-Like Syndrome in Aquarium *Echinopora lamellosa*

**DOI:** 10.1371/journal.pone.0097018

**Published:** 2014-05-14

**Authors:** David Smith, Peter Leary, Mark Bendall, Edmund Flach, Rachel Jones, Michael Sweet

**Affiliations:** 1 School of Biology, Newcastle University, Newcastle upon Tyne, Tyne and Wear, United Kingdom; 2 School of Biological Sciences, Queen’s University Belfast, Belfast, County Antrim, United Kingdom; 3 Zoological Society of London, London, Greater London, United Kingdom; 4 Biological Sciences Research Group, University of Derby, Derby, Derbyshire, United Kingdom; Catalan Institute for Water Research (ICRA), Spain

## Abstract

This study investigates potential causes of a novel blister-like syndrome in the plating coral *Echinopora lamellosa.* Visual inspections of this novel coral syndrome showed no obvious signs of macroparasites and the blisters themselves manifested as fluid-filled sacs on the surface of the coral, which rose from the coenosarc between the coral polyps. Histological analysis of the blisters showed that there was no associated necrosis with the epidermal or gastrodermal tissues. The only difference between blistered areas and apparently healthy tissues was the presence of proliferated growth (possible mucosal cell hyperplasia) directly at the blister interface (area between where the edge of the blister joined apparently healthy tissue). No bacterial aggregates were identified in any histological samples, nor any sign of tissue necrosis identified. We conclude, that the blister formations are not apparently caused by a specific microbial infection, but instead may be the result of irritation following growth anomalies of the epidermis. However, future work should be conducted to search for other potential casual agents, including viruses.

## Introduction

According to the UNEP Global Coral Disease Database [1], there are currently 27 types of disease reported to affect wild scleractinian corals throughout the world. These diseases include: coral bleaching, white plagues, white syndromes, yellow band diseases, black band disease, coral ciliate infections, fungal infections and growth anomalies. Most commonly, coral diseases are attributed to bacterial pathogens, such as cyanobacteria in Black Band Disease [2]–[5] and a *Vibrio* consortium in Yellow Band Disease [6], [7]. Other pathogenic agents in corals receive considerably less attention but include ciliates, associated with Brown Band Disease [8], [9], White Syndrome [8], Skeletal Eroding Band [10], [11]and the Caribbean Ciliate Infection [12], and fungal pathogens associated with Dark Spot Syndrome [13] and *Aspergillosis* in gorgonian sea fans [14]–[17].

Coral growth anomalies on the other hand have received even less attention. Work et al. [18] described the appearance of 8 varying types of growth anomaly existing in corals belonging to the genera *Acropora* alone, highlighting the importance of this syndrome to the health of corals. Predominantly, those studies which focus on this area of research appear to largely pertain to irregularities in skeletal growth [18]–[25], with few reports on associated hyperplasia and hypertrophy [18]–[20], [25]. Although focus on coral disease in the wild is currently high, research on aquarium coral diseases is severely lacking in the scientific literature, as highlighted by the recent review, Sweet et al. [26]. Although published reports of aquarium diseases are rare, there is a substantial amount of anecdotal reports of novel diseases by hobbyists and aquarium curators [27]. One such disease or syndrome is blistering of the epidermis. These blisters are thought to only affect the epidermis, appearing as raised ‘sacs’ (up to ∼1 cm^2^) on the surface of the coral. These ‘blisters’ often rupture, and a disease, similar in appearance to the wild type known as White Syndrome’ [26] occurs, often resulting in the loss of whole colonies.

This study, therefore, aimed to assess blistered *Echinopora lamellosa* corals and directly compare them to healthy *E. lamellosa* colonies kept in the same environment. We assessed these changes with histological sections of healthy tissues and tissues pertaining to the blister itself. Furthermore, as many coral diseases are associated with pathogenic microorganisms, we wanted to screen this syndrome for presence of any known coral pathogens.

## Methods

**Figure 1:**
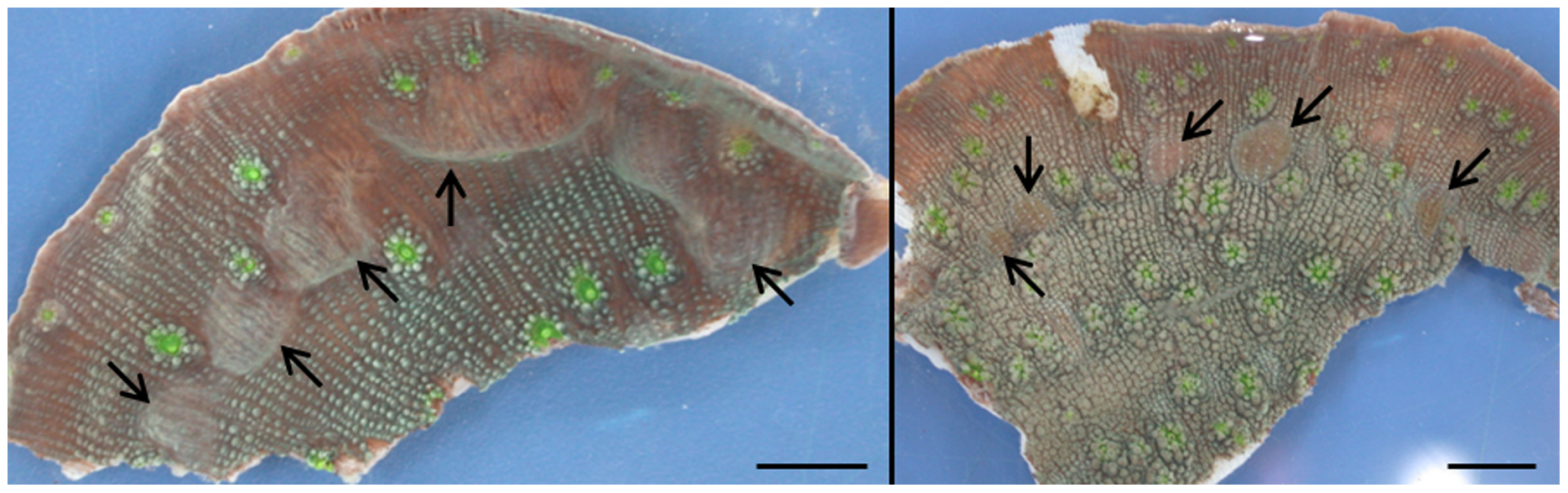
Photographs provided by the ZSL of *Echinopora lamellosa* fragments exhibiting blisters on the coral surface (highlighted by the arrows). Scale bars = 1 cm.

Specimens of *Echinopora lamellosa* present within the aquarium system at the Zoological Society of London (ZSL) were found to demonstrate the formation of blister-like mounds in surface tissue (Fig. 1). A preliminary pathological examination carried out at ZSL was inconclusive, with no evidence of bacteria or metazoa, but two types of ciliated protozoa were observed in direct microscopic preparations of affected tissue, and some fungal hyphae in a histological preparation. Further samples were divided and placed in 5% paraformaldehyde for histological analysis and 100% ethanol for microbial analysis (see below). Whole corals samples were collected and separated into healthy non-affected coral separate from the blistered coral (H); apparently healthy skeleton and tissue 1 cm away but adjacent from a blister (AH); and the blister (B) itself. AH and B tissues were sourced from only two colonies of *E. lamellosa* as these were the only corals which were showing signs of the syndrome. However, multiple replicates of the blister themselves on these corals were analysed within this study to allow for any variation between samples.

All coral samples used in this work were donated by the Zoological Society of London with the permission of the aquarium team.

### Histological Analysis

Samples for histological analysis (n = 5 for each sample type) were preserved in 50 ml falcon tubes containing 5% paraformaldehyde for 24 hr and subsequently stored in 100% EtOH before embedding in LR white resin [28]. B tissue sections were of the actual raised blister tissue itself. All tissue sections were cut to a thickness of 1 µm using a diamond knife on a RMC MT-XL ultramicrotome. H and B survey sections were stained using the general DNA stain toluidine blue [27]. To assess the extent of tissue necrosis, the stain nigrosin was applied to tissue sections [28]. Necrotic cells would appear black/brown in colouration; a lack of staining indicates tissues are not necrotic. Acridine orange, utilised to differentiate live/dead tissues and localise any microorganisms was also used [29], [30]. Positive controls were carried out for each stain by applying them to sections from other diseased scleractinian corals that were positive for bacterial aggregates, as well as tissue necrosis (Figure S1). Sections were viewed at magnification x 1000 using a Leica DMRB light microscope and images taken using an integrating camera (QICAM Fast 1394).

### DNA Extraction and Microbial Analysis

To determine microbial community shifts in response to blister formation on the surface of *Echinopora lamellosa*, n = 3 samples for each sample type (H, AH and B) were analysed. Upon sampling, coral fragments were stored in 50 ml falcon tubes filled with 100% Ethanol and kept at 4°C until fragments were to be individually crushed using a sterilised pestle and mortar. DNA of whole coral samples was extracted using the QIAGEN DNeasy Blood and Tissue Kit and resultant extracted DNA stored at 4°C.

Bacterial 16 S rRNA gene diversity was assessed using the universal bacterial primers 357F (5′-CCTACGGGAGGCAGCAG-3′) and 518R (5′ATTACCGCGGCTGCTGG-3′) following the procedure by Sweet et al. [31] Ciliate-specific 18S rRNA gene amplification was carried out using the same ciliate primers as Sweet and Bythell [8]; forward primer CilF (5′-TGGTAGTGTATTGGACWACCA-3′) with a 36 bp GC clamp [32] attached to the 5′ end and the reverse primer CilDGGE-r (5′-TGAAAACATCCTTGGCAACTG-3′). ITS fungal 18S rRNA gene was amplified using a nested approach as in Sweet et al. [13]. First the primers ITS1F (5′-CTTGGTCATTTAGAGGAAGTAA-3′) and tITS4 (5′-TCCTCCGCTTATTGATATGC-3′) were used in the initial round of PCR. Followed by primers ITS3F (5′-GCATCGATGAAGAACGCAGC-3′) and ITS4-GC (5′-CGCCCGCCGCGCCCCGCGCCCGGCCCGCCGCCCCCGCCCCTCCTCCGCTTATTGATATGC-3′). All protocols and PCR conditions were the same as Sweet et al. [13]. Algal 18S rRNA gene was amplified using the same un-nested approach as Zhao et al. [33]. The forward primers developed by Diez et al. [34]; Euk1A (5′-CTGGTTGATCCTGCCAG-3′) and the reverse primer Euk516r-GC (5′CGCCCGGGGCGCGCCCCGGGCGGGGCGGGGGCACGGGGGGACCAGACTTGCCCTCC-3′) were used in PCR amplification. PCR reaction mixtures contained 10 ng of extracted template DNA, incubation buffer (MP Biomedicals), 2 mM MgCl_2_ (MP Biomedicals), 0.25 mM dNTP (QIAGEN), 0.5 mM of each primer and 2.5 U of DreamTaq™ DNA Polymerase (Fermentas). PCR amplification consisted of primary denaturation being carried out at 95°C for 2 min, followed by 35 PCR cycles at 95°C for 30 sec, 50°C for 30 sec and 72°C for 2 min, 30 sec. A final elongation step at 72°C was then carried out for 7 min. All PCR reactions listed above were carried out using a Hybaid PCR Express thermal cycler. Controls for all samples were run alongside the coral samples to ensure the PCR was successful.

DGGE was performed using the D-code universal mutation detection system (Bio-Rad). Bacterial 16S rRNA and fungal PCR products were resolved on 10% (w/v) polyacrylamide gels using a 30 to 60% denaturant gradient, while ciliate and microalgae PCR products were resolved on 6% (w/v) polyacrylamide gels using a 32–42% denaturant gradient. DGGE was carried out for 16 hours at 60°C, with a constant voltage of 50 V. Gels were stained as per Sweet et al. [35]. To identify bands of most interest in the DGGE gels (those that represented the greatest differences/similarities between samples), representative bands were excised from the gel, left overnight in Sigma molecular grade water and then re-amplified using primers 357F and 518R. The products of each re-amplified band were then verified by agarose gel electrophoresis [1% (w/v) agarose] with ethidium bromide staining and visualised using a UV transilluminator. PCR products were then purified using the QIAGEN PCR Purification Kit (see main methods section for complete description) and then labelled using a Big Dye (Applied Biosystems) transformation sequence kit and sent to Genevision (University of Newcastle) for sequencing. Sequences were then compared with sequences in the BLAST nucleotide database (NCBI) to allow for genus/species matching. Any heteroduplexes identified following repeated sequence alignments in BLAST [36] were removed from the data.

Using Bionumerics 3.5 (Applied Maths BVBA) software, bacterial operation taxonomic units (OTUs) were defined from DGGE band-matching analysis. Standard internal marker lanes were used to allow for gel-to-gel comparisons. Tolerance and optimisation for band-matching was set at 1%.

### Statistical Analysis

Relative bacterial operation taxonomic units (OTUs) were defined from DGGE band matching analysis using BioNumerics 3.5 (Applied Maths BVBA) software. Tolerance and optimisation for band matching was set at 1%. For DGGE profiles, a pairwise ANOSIM based on Bray-Curtis indexes were performed to determine differences between gene assemblages associated with the different coral samples (H, AH, and B). In order to determine which bacterial ribotypes showed the greatest shifts in presence between sample types, similarity percentages (SIMPER) analyses were conducted. All statistical analyses on bacterial DGGE profiles were conducted using PRIMER V. 6 software [7], [8], [37], including a multidimensional scaling (MDS) plot based on Bray-Curtis similarities.

## Results

### Visual Assessment

Visual inspection of the blisters carried out at the Zoological Society of London showed that the blisters varied in size from between 2 and 8 mm. Following visual inspection under dissecting microscope, blisters routinely occurred within the coenosarc and there were no visual signs of any macro organisms associated with the blistered tissue or the coral skeleton. The blisters were fluid filled sacs which contained coral mucus in some instances.

### Histology of Healthy and Blister-affected Tissues

**Figure 2:**
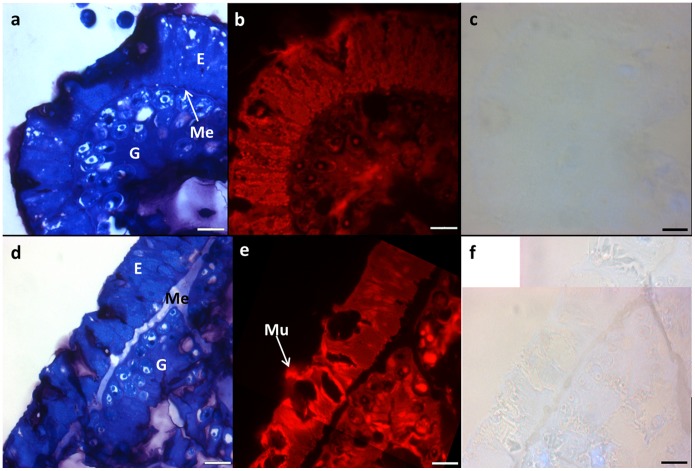
Representative histological sections from healthy (a, b and c) and blister-affected (d, e and f) *Echinopora lamellosa* tissues embedded in LR white resin. Blister sections were taken at the blister-affected interface. A) and d) Survey sections stained with toluidine blue (E = epidermis, G = gastrodermis and Me = mesoglea). B) and e) Sections stained with acridine orange for the detection of bacteria, as visualised by red fluorescence. In this case, red fluorescence is attributed to autofluorescence of symbiotic algae nuclei and coral mucus (Mu). C) and f) Nigrosin staining, targeting necrotic tissues. Scale bars = 10 µm.

**Figure 3:**
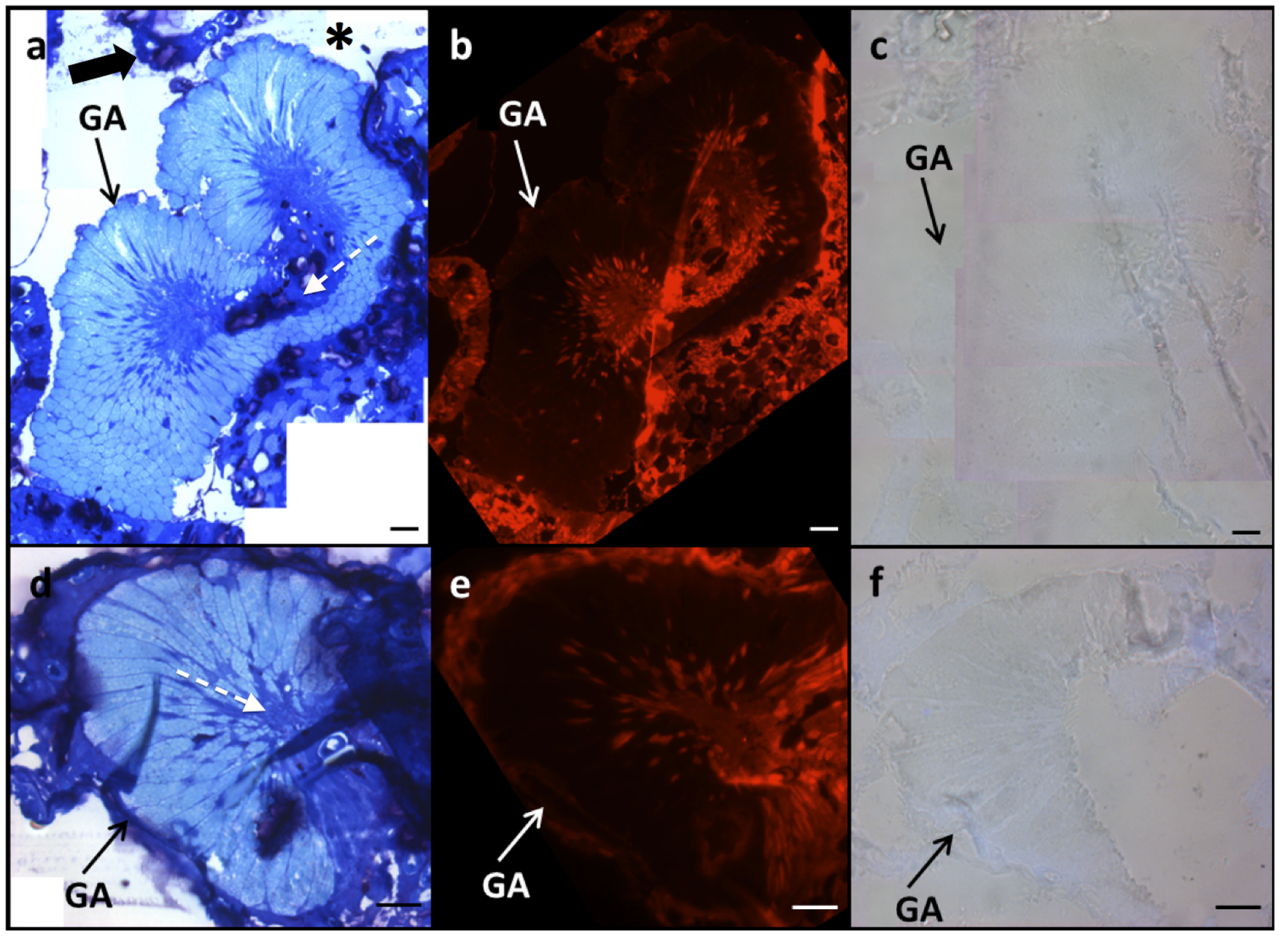
Histological sections of growth anomalies (GA) only identified in blister-affected samples embedded in LR white resin. A) and d) Survey sections stained with toluidine blue. B) and e) Sections stained with acridine orange for the detection of bacteria, as visualised by red fluorescence. In this case, red fluorescence is attributed to autofluorescence of host nuclei (e.g. mucocyte nuclei). C) and f) Nigrosin staining, targeting necrotic tissues. Block arrow indicates epithelium separated from underlying tissue, revealing a cleft (represented by the asterisk). Dashed arrow highlights growth from a centralised area. Scale bars = 10 µm.

Survey sections stained with toluidine blue demonstrate no visual differences in the surface tissues of healthy and blister-affected samples (Fig. 2a, 2d), with the epidermis (mucocyte layer) and gastrodermis (zooxanthellae layer) appearing normal and intact in both sample types. The main difference between sample types was the presence of growth anomalies (GA) within all survey sections of blister-affected samples (Fig. 3a, 3d). GAs appear to be morphologically similar to the coral mucocyte layer, with minute vesicles (∼0.5–1 µm in length) appearing therein and striations within the layer (Fig. 2a, 2d, 3a, 3d). Growth appeared to start from a centralised area (dashed arrow) and proliferate outwards (illustrating a possible hyperplasia), filling free spaces and pressing up against apparently healthy tissue (Fig. 3a, 3d). Furthermore, separation of the epithelium from the underlying tissue revealing a cleft was apparent, likely resulting in the formation of a blister (Fig. 3a). Sections stained with acridine orange only exhibit fluorescence in the nuclei of zooxanthellae and host cells, as well as at sites of coral mucus (Figs. 2b, 2e, 3b, 3e). No tissue sections were found to test positive for signs of tissue necrosis (Fig. 2c, 2f, 3c, 3f).

### Variation in Microbial Diversity

There was no significant difference in 16S rRNA diversity between sample types (Pairwise ANOSIM; H and AH, R = 1, *p* = 0.1. H and B, R = 1, *p* = 0.1. AH and B, R = 0.852, *p* = 0.1). Only two bacterial ribotypes were detected in blister-affected samples that were found to be completely absent in healthy and apparently healthy samples, including a ribotype similar to a *Bacterioides* species (GenBank accession number NR041446) and a *Moritella* species (NR028880). In contrast seven ribotypes were found to be associated with the healthy holobiont, but increased in apparently healthy and blister samples. This includes ribotypes related to; a *Bacteroides* species (NR041446), a *Bradyrhizobium* species (CP000494), a *Halobacteriacae* species (NR102920), a *Novosphingobium* species (NR074261), a *Pseudoalteromonas* species (NR026221), a *Shewanella* species (NR044863) and a *Tenacibaculum* species (NR044498), with the *Bradyrhizobium* species (CP000494) showing a relative increase (in relation to DGGE band intensity) in apparently healthy and blister tissues. Certain ribotypes were found to be reduced or absent in apparently healthy and blister samples compared to healthy samples. This includes ribotypes closely related to: an *Arcobacter* species (NR044549), a *Chloroflexus* species (NR102515), a *Desulfotomaculum* species (NR027608), a *Mesorhizobium* species (NR102452), a *Pseudomonas* species (NR102835), a *Vibrio* species (NR102976) and a species of *Bacteroidetes* (GQ274078). Another ribotype related to an *Arcobacter* species (NR102873) along with ribotypes related to a *Cyanothece* species (NR074265) and a *Lactobacillus* species (NR043182) were present across all samples, maintaining relatively high abundance in all sample types.

There were no detectable ciliates or fungi associated with either the healthy, apparently healthy or blister-affected *Echinopora lamellosa* samples following molecular analysis. The only microalgae, detected within this study were found to belong to *Symbiodinium sp.*, the zooxanthellae normally associated with scleractinian corals. No invasive or pathogenic microalgae were found.

## Discussion

This is the first study to describe a blister-like syndrome in the scleractinian coral *Echinopora lamellosa*. Histological analysis showed the epidermis of blister-affected samples was found to be similar to that of healthy samples, with the corals Symbiodinium, appearing to remain intact and blistered tissues showing no signs of necrosis. Given that acridine orange only appeared to show autofluorescence of coral, zooxanthellae and coral mucus, there were no microbial associates present within the blistered tissues, highlighting that in contrast to many other coral diseases [2]–[8], [10], [13]–[17], [27], [58], [59], this syndrome is unlikely caused by microbial pathogens. Instead, all blistered sections exhibited growth anomalies of the coral tissue (GAs) that appeared morphologically similar to the mucocyte layer of the epidermis. The main visual difference between GAs and the regular epidermis is that the GAs appear to grow outwards from a central point, with GAs possibly being an occurrence of hyperplasia in mucosal cells. This could potentially compromise healthy tissue as contact is made, resulting in irritation of the tissues and the formation of the blisters themselves. Based on comparative staining and comparisons with healthy tissue, GAs appear to show small aggregations of mucus, a multitude of tiny vesicles and striations within the tissue, similar to that found in association with mucocytes in regular coral epidermis. The growth anomalies seen here in *E. lamellosa* differ from those reported in most other scleractinian corals, with reports of previous growth anomalies often including proliferated skeletal growth, resulting in nodular, exophytic calices [38]–[44]. These reports also state that tissue overlying skeletal growth anomalies is translucent (lacking zooxanthellae), whereas the blisters in this study maintain the same pigmentation to the surrounding apparently healthy tissue (with zooxanthellae appearing normal in histological sections of blister-affected tissues). Growth anomalies of the encrusting coral *Montipora efflorescens* have differed from this, being described as low-lying, umbonate growths (based on nomenclature for describing growth anomalies outlined by Work and Aeby [45]), but still pertain to the coral skeleton [38]. The growth anomalies found in *E. lamellosa* do not appear to include exophytic growth of the underlying skeleton. Hyperplasia has previously been associated with growth anomalies in scleractinian corals, with previous reports identifying hyperplasia in the basal body wall [38], [42]. Again, this differs to the observed hyperplasia in *E. lamellosa* in this study, with hyperplasia appearing to take place in the epidermal layer, potentially occurring in mucosal cells. Furthermore, hyperplasia in the encrusting coral *Montipora spp* has been found to be associated with microalgae in some instances [38]. However no invasive microalgae were identified in *E. lamellosa* exhibiting growth anomalies. Interestingly, reports of hyperplasia in soft corals on the other hand, has been found to take place in the coenenchyme, between polyps (the region in which blisters were found to occur here in *E. lamellosa*), but in soft corals, Williams et al [38] also found a distinct necrotizing component associated with growth anomalies (not found in *E. lamellosa*).

Assessing the bacterial communities associated with healthy corals and their subsequent loss in response to a stress event or disease could highlight potential probiotic bacterium associated with the health of the holobiont [46]. In this study, the dominant prokaryotic community was not found to shift significantly between different health states, which is in contrast to other coral disease studies [8], [47]–[49]. Only four bacterial ribotypes were present in healthy *E. lamellosa* samples and absent in both the apparently healthy tissues and the blistered samples. These included ribotypes closely related to a *Desulfotomaculum* species, a *Mesorhizobium* species, a *Chloroflexus* species and a *Pseudomonas* species. The *Desulfotomaculum* and *Mesorhizobium*-related species had relatively high abundance in healthy samples and thus a loss of these species from the coral-associated community may be of particular importance to coral health and function within the holobiont. Interestingly, *Desulfotomaculum spp* are spore forming sulphate-reducing bacteria that have previously been associated with black band disease in scleractinian corals [50], [51], despite their association with only healthy tissue in this study. A *Mesorhizobium sp* has been associated with healthy scleractinian corals in the past [52], with this bacterium considered to have a symbiotic nitrogen-fixing relationship within the host coral [53]. Furthermore, Littman et al. [54] found *Mesorhizobium sp* to be specifically associated with the corals algal symbionts, the *Symbiodinium* that reside within the host coral, indicating that this symbiotic nitrogen-fixing association may actually be formed specifically between the *Symbiodinium* and the bacteria *Mesorhizobium*.

Although there is yet to be any conclusive evidence linking viral infections with GAs in scleractinian corals, viruses have been associated with other diseases and stresses in scleractinian corals in the past [55]; including environmentally stressed corals [56], bleaching [57], [58] and most recently, white plague disease [58]. Added to the association of viruses implicated in tumour formation in other marine organisms [59], including fibropapillomatosis in marine turtles [60]–[63] and a plethora of fish tumours [64], [65], there exists the potential that GAs in scleractinian corals may be the result of a viral infection [55], [66], [67]. To date, there are few coral disease studies that have addressed the role viruses in coral health and disease and future work should accommodate for this gap in the field of coral disease research.

Finally, although we were unable to test for this fact, the coral skeleton of blister-affected specimens was found to be softer than that of healthy samples (assessed when crushing samples for microbial analysis). This suggests the structure of the coral skeleton may be impaired, with similar finds (such as a finer skeletal structure and lower skeletal density) being reported in other scleractinian corals exhibiting growth anomalies [42]–[44], [68]. Thus, focus on skeletal effects of coral blisters warrants further investigation. To conclude, this is the first study to highlight the appearance of soft tissue growth anomalies in aquarium corals and highlights that the syndrome is unlikely to be the result of a microbial pathogenic agent (bacteria, fungi, protozoan or algae). More likely the blister formation appears to be the result of growth anomalies resulting from irregular epidermal growth. As these blisters have been shown to be a precursor to instances of diseases such as White Syndrome, there is an obvious need to increase our understanding of this syndrome in both aquarium systems and in the wild. Future work should focus on further isolation and understanding of similar blister-like pathologies in other scleractinian coral species.

## Supporting Information

Figure S1 **Histological sections of scleractinian corals positively stained with nigrosin and acridine orange, respectively.** A) Section of a scleractinian coral stained with nigrosin to reveal necrosis in coral associated zooxanthellae (highlighted by black arrow). B) Section of a scleractinian coral stained with acridine orange to reveal bacterial aggregates (white arrows). Scale bars = 10 µm.
(TIFF)
