# Supplementary figures and images for: A Novel Investigation of a Blister-Like Syndrome in Aquarium Echinopora lamellosa
Source: PLoS One. 2014 May 14;9(5):e97018. doi: 10.1371/journal.pone.0097018 (PMC4020768; doi:10.1371/journal.pone.0097018)

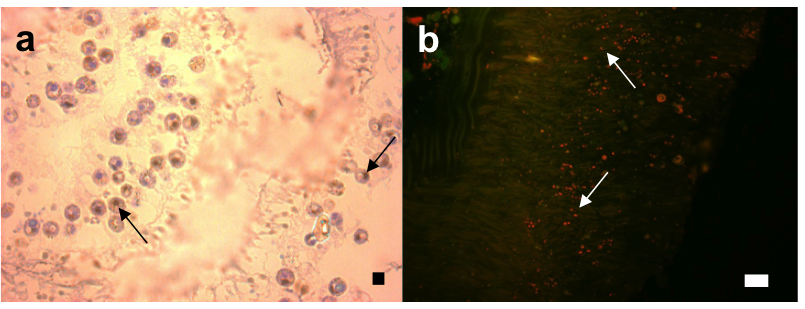

Supplement: Figure S1 — Histological sections of scleractinian corals positively stained with nigrosin and acridine orange, respectively. A) Section of a scleractinian coral stained with nigrosin to reveal necrosis in coral associated zooxanthellae (highlighted by black arrow). B) Section of a scleractinian coral stained with acridine orange to reveal bacterial aggregates (white arrows). Scale bars = 10 µm. (TIFF) [file pone.0097018.s001.tiff]
